# Real-world insights and outcomes related to ciclosporin A 0.1% cationic emulsion for the long-term treatment of dry eye disease in Germany: Country-level sub-analysis of the PERSPECTIVE study

**DOI:** 10.1007/s00417-024-06414-z

**Published:** 2024-05-09

**Authors:** Ines Lanzl, Christoph M E Deuter, Katrin Lorenz, Gerd Geerling, on behalf of the PERSPECTIVE German study group

**Affiliations:** 1 Chiemsee Augen Tagesklinik, Geigelsteinstrasse 26, 83209 Prien, Germany; 2 Department of Ophthalmology, Technical University of Munich, Munich, Germany; 3 Centre for Ophthalmology, Eberhard-Karls-University, Tübingen, Germany; 4 Department of Ophthalmology, University Medical Center, Johannes Gutenberg-University Mainz, Mainz, Germany; 5 Department of Ophthalmology, University Hospital Düsseldorf, Düsseldorf, Germany

**Keywords:** Ciclosporin A 0.1% cationic emulsion, Dry eye disease, Real-world evidence, PERSPECTIVE study, Severe keratitis, Germany

## Abstract

**Purpose:**

The PERSPECTIVE study was a real-world European, non-interventional, multicenter, observational study that evaluated the effectiveness, tolerability, and safety of ciclosporin A (CsA) 0.1% cationic emulsion (CE) in routine clinical practice as a treatment for adults with severe keratitis and dry eye disease (DED) that remained insufficiently controlled with artificial tears. This sub-analysis examined data from ophthalmology clinics in Germany to provide more precise insights into treatment patterns, outcomes, and clinical decision-making related to CsA 0.1% CE.

**Methods:**

Study data were collected from adults starting CsA 0.1% CE (one drop in both eyes at bedtime) and followed up at Week 4, 12, and 24, and Month 12. The primary endpoint was mean change from baseline in corneal fluorescein staining (CFS) score (Oxford Grade Scale) at Month 12. Secondary endpoints examined the severity of ocular signs and symptoms, and adverse events (AEs).

**Results:**

A total of 236 patients from 20 ophthalmology clinics in Germany participated in the PERSPECTIVE study (69.9% female; mean age 60.8 years). Following treatment with CsA 0.1% CE, patients experienced significant reductions in CFS score from Week 4, which were maintained through to Month 12 (*P* < 0.0001). From baseline, 81.6% of patients experienced an improvement in CFS score at Month 12. CsA 0.1% CE provided significant reductions in the severity of eyelid and conjunctival erythema at Month 12 compared with baseline (*P* < 0.001), as well as significant reductions in the severity of subjective ocular symptoms (all *P* ≤ 0.015). Safety data were consistent with the known safety profile of CsA 0.1% CE. Tolerability was rated as “satisfactory,” “good,” or “very good” by 97.2% of physicians and 95.7% of patients.

**Conclusion:**

Outcomes in Germany were similar to those reported for the overall European study population and are indicative of the treatment results that ophthalmologists may expect to see with CsA 0.1% CE treatment in real-life clinical practice. Treatment with CsA 0.1% CE provided long-term improvements over 12 months and was generally well tolerated.


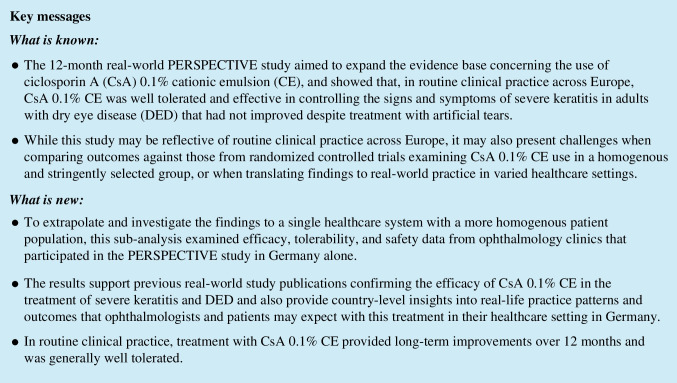


## Introduction

Dry eye disease (DED), also known as keratoconjunctivitis sicca, is a multifactorial, complex, and chronic ocular condition, requiring long-term treatment, which may be challenging to diagnose and treat in clinical practice [1–6]. The condition is fairly common, with epidemiological data in Europe showing a prevalence ranging from 11 to 30% [4]. It is defined by the Tear Film Ocular Surface Society (TFOS) Dry Eye Workshop (DEWS) II as a “multifactorial disease of the ocular surface characterized by a loss of homeostasis of the tear film, and accompanied by ocular symptoms, in which tear film instability and hyperosmolarity, ocular surface inflammation and damage, and neurosensory abnormalities play etiological roles” [1]. Symptoms include ocular irritation, foreign body sensation, photophobia, eye pain, and impaired/blurred vision, as well as itching, burning/stinging, and/or scratching sensations, all of which can limit performance of daily tasks and quality of life [1, 2, 5].

DED is typically classified as either evaporative or aqueous deficient [1]. Meibomian gland dysfunction is one of the underlying causes of evaporative DED, whereas tear underproduction results in aqueous-deficient dry eye [1]. However, most people with DED display signs and symptoms in varying combinations that relate to both types of disease [1]. It is a progressive condition, resulting in inflammation and damage to the ocular surface, caused, in part, by increased osmolarity of the tear film [6]. Regardless of whether the underlying mechanism for DED is increased tear evaporation or aqueous deficiency, or both, the homeostasis of the ocular surface is disrupted and pro-inflammatory pathways are activated, causing ocular surface damage and neurosensory aberrations, leading to a vicious and self-perpetuating cycle of inflammation and worsening pathophysiology [1, 2, 7–9]. DED is typically cyclical in nature with episodic worsening (or flares) of signs and symptoms occurring over time, including prolonged discomfort and ocular inflammation (usually indicated by the presence of eyelid and/or conjunctival hyperemia) [5]. If left untreated, DED may progress in severity and result in permanent ocular damage [5].

Once a diagnosis has been reached, management of DED focuses on restoring both the tear film and ocular surface homeostasis, treating the underlying pathophysiology, preventing symptomatic flares, and reducing complications [1, 2, 8]. Management of DED typically follows a stepwise approach, beginning with education on the disease and behavior modification (including diet, environment, lid hygiene, and application of warm compresses), as well as the use of tear substitutes or artificial tears (ATs) [8]. ATs protect and lubricate the ocular surface but do not tackle the underlying pathogenesis and inflammation associated with the condition. DED progresses in most cases, requiring the addition of topical anti-inflammatory treatments, such as corticosteroids or ciclosporin, to reduce inflammation and help improve quality of life [2, 3, 5, 8, 10–17]. If DED progresses, it is advised by the TFOS DEWS II and German Ophthalmology Society (DOG) guidelines, and recent expert consensus recommendations, that anti-inflammatory treatments should be initiated as early as possible, when the ocular surface is likely to be responsive to therapy (such as when first-line ATs no longer adequately control symptoms) [4, 5, 8, 18]. Corticosteroids are effective at controlling inflammation, but their long-term use is associated with a risk of ocular complications, such as ocular hypertension (or glaucoma), cataracts, and infections [8, 19]. In contrast, topical ciclosporin A (CsA) is a widely used anti-inflammatory treatment that can be administered over the long term and is approved for the treatment of severe keratitis in patients with DED that has not improved despite treatment with ATs [20]. Topical CsA is available as either a 0.05% anionic or 0.1% cationic emulsion (CE); however, CsA 0.1% CE ophthalmic solution is the only commercially available treatment in Europe [20]. Clinical studies have established the efficacy and tolerability of CsA 0.1% CE and report a reduction in corneal surface damage and ocular surface inflammation with up to 24 months of follow-up [11–15]. Three real-world studies have reported similarly improved outcomes with CsA 0.1% CE in patients with DED [5, 16, 21], including a recent real-world experience report from the UK which showed that patients with ocular surface inflammatory diseases (particularly those with DED) experience improved clinical outcomes with CsA 0.1% CE treatment and a reduced need for adjunctive steroids [16].

The 12-month real-world PERSPECTIVE study showed that, in routine clinical practice across Europe, CsA 0.1% CE was well tolerated and effective in controlling the signs and symptoms of severe keratitis in adults with DED that had not improved despite treatment with ATs [5]. This study was designed to reflect real-world clinical practice and specified that treatment should be prescribed in accordance with the approved label for CsA 0.1% CE, as judged by the study investigator, rather than specifying a formal threshold corneal fluorescein staining (CFS) score or the presence of any specific signs and symptoms of DED [5]. This large, multicenter, observational study reported that CsA 0.1% CE significantly reduced ocular surface damage and improved the severity and signs of DED from Week 4 through to Month 12, and provides insights into outcomes that ophthalmologists and patients may expect with this treatment [5].

Since the PERSPECTIVE study included various European patient populations and allowed patients to enter the study if judged by the investigating clinician to have severe keratitis and DED (which may differ based upon a combination of factors), it may include a diverse and heterogenous study population [5]. For example, although the approved label for CsA 0.1% CE states that it should be used in the treatment of severe keratitis and DED, no formal threshold for CFS score (using the Oxford Grade Scale) is stipulated in the licensed indication, and the literature in this area does not clearly define the way in which disease severity should be graded [1, 4, 7, 8, 18, 20]. Therefore, in routine clinical practice, ophthalmologists may evaluate the severity of keratitis and DED using a combination of CFS score, other signs (e.g., eyelid and/or conjunctival erythema), patient-reported symptoms, and quality of life factors [5]. This approach reflects the complex and multifactorial nature of the disease. While the approach in the PERSPECTIVE study may be reflective of routine clinical practice across Europe, it may also present challenges when comparing outcomes against those from randomized controlled trials (RCTs) examining CsA 0.1% CE use in a homogenous and stringently selected group [5], or when translating findings to real-world practice in varied healthcare settings.

To extrapolate and investigate the findings to a single healthcare system with a more homogenous patient population, the current sub-analysis examined efficacy, tolerability, and safety data from ophthalmology clinics that participated in the PERSPECTIVE study in Germany [5]. This can provide more precise insights into treatment patterns, outcomes, and clinical decision-making related to CsA 0.1% CE in Germany, and will detract from any differences between real-world approaches in the management of DED between countries based on the healthcare system and providers, reimbursement criteria, variations in access to healthcare, local guidelines, and/or variations in physician assessments and evaluation of disease severity. This sub-analysis will also provide an opportunity to explore the areas of commonality and contrast between the data from Germany and other regional data regarding treatment outcomes achieved, and the patient populations considered by the treating physicians to have disease of sufficient severity to warrant CsA 0.1% CE treatment, in routine clinical practice. To our knowledge, this sub-analysis includes the largest cohort of patients treated for DED in Germany.

## Methods

The PERSPECTIVE study comprised a 12-month, European, prospective, non-interventional, multicenter, observational study of adults (≥ 18 years of age) with severe keratitis and DED, currently treated with ATs. In line with the European Medicines Agency (EMA) requirements, the study was registered under the European Network of Centres for Pharmacoepidemiology and Pharmacovigilance (ENCePP^®^) European Union electronic Register of Post-Authorisation Studies (EU PAS Register) (EU PAS Register Number: EUPAS22376). All patients included were required to provide written informed consent prior to their enrollment. The centers/institutions are listed alongside the relevant investigator in the PERSPECTIVE Germany study group section at the end of this article. The current sub-analysis examined PERSPECTIVE study data collected from 20 ophthalmology clinics in Germany. The study methodology was previously reported [5], but the key elements are included below.

Data were collected between 11 April 2017 and 14 November 2019 during routine appointments at participating ophthalmology clinics in Germany at baseline and then following CsA 0.1% CE initiation at Week 4, 12, and 24, and Month 12. Attendance at baseline and Month 12 was mandatory for inclusion in the study, and data were reported for patients choosing to attend interim study visits. Baseline measures were recorded under AT treatment, and the eye with the highest baseline CFS score (Oxford Grade Scale; Grade 0–V) was chosen as the study eye. Variables were documented for each eye separately at baseline and subsequent study visits. In cases where the CFS score was equal in both eyes at baseline, the right eye was selected as the study eye. As the study was conducted in a routine clinical practice setting, all study medications (CsA 0.1% CE, ATs, or corticosteroids) were prescribed and reimbursed, or paid for, in accordance with local healthcare arrangements. No medication was supplied by the study sponsor.

### Study population

Patients were selected for inclusion in the study by ophthalmologists practicing in Germany according to the approved CsA 0.1% CE label [20]. Eligible patients for study entry were ≥ 18 years of age with a diagnosis of severe keratitis and DED, and currently receiving AT treatment. Patients were excluded from the study if they had received, or were currently receiving, treatment with CsA 0.1% CE, were taking immunosuppressant therapy, had previous/planned eye surgery (within 6 months), or were pregnant, breastfeeding, or planning a pregnancy.

### Study treatment

Patients enrolled in the study received CsA 0.1% CE (one drop daily) in both eyes at bedtime for 12 months. Concomitant use of ATs and/or corticosteroids was allowed.

### Study endpoints

The primary endpoint was mean (standard deviation [SD]) change from baseline in CFS score (Oxford Grade Scale; Grade 0–V) at Month 12 following CsA 0.1% CE initiation. Secondary endpoints were change from baseline in CFS score at each study visit; change from baseline in severity of eyelid erythema and conjunctival erythema (measured using a 4-point scale: none, mild, moderate, or severe); change from baseline in visual acuity (VA; decimal scale), Schirmer’s test (without anesthesia) and tear break-up time (TBUT; seconds); and change from baseline in severity of ocular symptoms (measured using a 4-point scale: none, mild, moderate, or severe). Schirmer’s test and TBUT assessments were optional at study visits. Ocular symptoms assessed during the study comprised photophobia, blurred vision, sticky feeling, eye pain, itching, burning/stinging, and foreign body sensation. Symptom severity was recorded using a 4-point scale in each case (none, mild, moderate, or severe). VA data were collected using either decimal, fraction (feet or meters), or logMAR (logarithm of the minimum angle of resolution) scales. All values were converted into decimal scale (the standard in many European countries) for analysis using appropriate conversion charts [22].

Investigators provided their evaluation of the effectiveness of CsA 0.1% CE and clinical signs during therapy with CsA 0.1% CE by comparing against prior treatment using a 3-point scale (better, the same, or worse). The prior treatment received by the patients differed according to routine practice, but remained within the approved CsA 0.1% CE label, and included ATs and/or corticosteroids. As the study was conducted in a routine clinical practice setting, all study medications were prescribed in accordance with local healthcare arrangements. No noticeable differences were noted between centers.

Patients and physicians reported their assessment of tolerability with the study medication using a 4-point scale (very good, good, satisfactory, or poor). As this was an observational study, carried out in a routine clinical practice setting, no validated questionnaire for patient assessment was mandatory for use. Adverse events (AEs) and treatment-related AEs were collected and documented at each visit and for the total study period.

### Statistical analysis

ICON Plc (Dublin, Ireland) conducted all statistical analyses on behalf of the PERSPECTIVE German study group. Results are presented for the full analysis set (FAS). Statistical analyses compared treatment outcomes at each study visit with baseline levels. The Bhapkar test was used to assess change in CFS score at Month 12 and interim visits, as well as change in the severity of symptoms, eyelid erythema, and conjunctival erythema. The Bhapkar test can be used in marginal homogeneity, and it assumes that the changes are non-directional [23]. A paired *t*-test or a Wilcoxon signed-rank test was used to assess the statistical significance of the change in CFS, VA, TBUT, and Schirmer’s test compared with baseline.

## Results

**Fig. 1:**
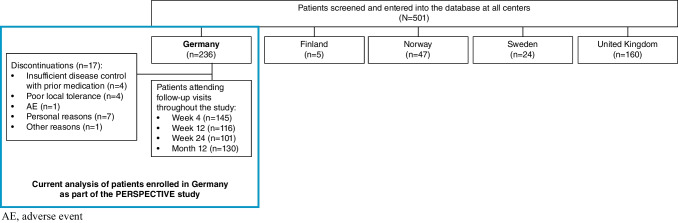
Flowchart of patient disposition

Of the 501 patients enrolled in the PERSPECTIVE study, 236 patients were enrolled in Germany (Fig. 1). Of these, 69.9% were female, and the mean age was 60.8 (SD 15.8; range 19.9–93.2) years. Table 1 summarizes the baseline patient and clinical characteristics. The main reasons reported for initiating CsA 0.1% CE treatment were insufficient control of keratitis or DED with prior medication (72.9%) and progression of keratitis or DED (28.4%). Physicians were allowed to provide more than one reason for initiating CsA 0.1% CE treatment.

**Table 1:** Baseline patient and clinical characteristics, and AT use (FAS population)

Study eye (worst CFS score at baseline), *n* (%)	
Left	35 (14.8)
Right	201 (85.2)
CFS score (Oxford Grade Scale), *n* (%)	
0	2 (0.9)
I	35 (15.0)
II	94 (40.2)
III	80 (34.2)
IV	22 (9.4)
V	1 (0.4)
Mean (SD) CFS score (*n* = 234)	2.38 (0.9)
AT use, *n* (%)	
6 times per day	18 (4.2)
5 times per day	75 (17.3)
4 times per day	49 (11.3)
3 times per day	77 (17.8)
Twice per day	24 (5.5)
Daily/once	113 (26.1)
Three times per week	1 (0.2)
As needed	37 (8.5)

*AT*, artificial tear; *CFS*, corneal fluorescein staining; *FAS*, full analysis set; *SD*, standard deviation

### Change in CFS score

At baseline, the mean CFS score was 2.38 (SD 0.90) and was typically Grade II (40.2%) or Grade III (34.2%) on the Oxford Grade Scale. The mean CFS score at the end of the study (Month 12) was 0.85 (SD 0.94). This reduction in mean (± SD) score of 1.58 (± 1.08) was statistically significant (*P* < 0.0001). Significant reductions in CFS score were also achieved at Week 4, Week 12, and Week 24 (all *P* < 0.0001; Table 2).

**Table 2:** CFS score at each study visit

	*n*^a^	Mean (SD)	*n*^b,c^	Change from baseline, mean (SD)	*P* value^c^
Baseline	234	2.38 (0.90)			
Week 4	125	1.67 (1.05)	125	0.79 (0.88)	< 0.0001
Week 12	107	1.46 (1.04)	107	1.00 (0.98)	< 0.0001
Week 24	96	0.96 (0.97)	96	1.46 (0.97)	< 0.0001
Month 12	123	0.85 (0.94)	122	1.58 (1.08)	< 0.0001

^a^Number of patients with a CFS measurement at each study visit

^b^Number of patients with a CFS measurement at baseline and at each subsequent study visit, used to calculate the change

^c^Significant testing using two-sided test

*CFS*, corneal fluorescein staining; *SD*, standard deviation

**Fig. 2:**
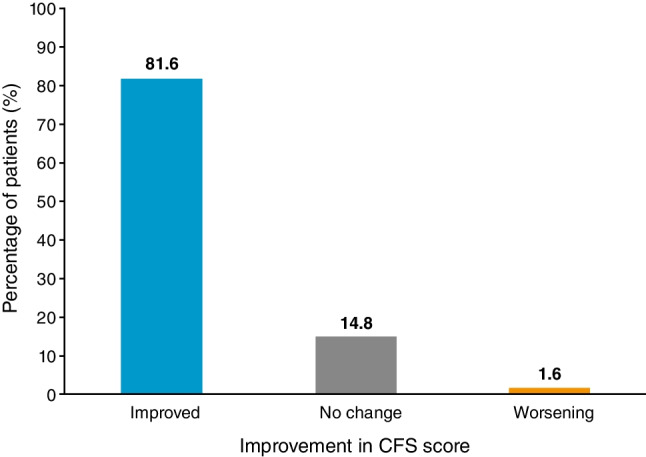
Change from baseline in CFS score at Month 12 following the initiation of CsA 0.1% CE. CE, cationic emulsion; CFS, corneal fluorescein staining; CsA, ciclosporin A

Over the 12-month period, an improvement in the CFS score was observed, with a shift in mode from a CFS grade II at baseline to CFS grade I at Week 4 and Week 12, CFS grade 0 and I at Week 24, and CFS grade 0 at Month 12. At Month 12, most patients (81.6%) demonstrated an improvement in CFS score following initiation of CsA 0.1% CE, 18 patients (14.8%) experienced no changes, and worsening occurred in two patients (1.6%), compared with baseline (Fig. 2). At Month 12, 20.5% of patients achieved an improvement in CFS of ≥ 3 grades from baseline. The percentage of patients with an improvement of ≥ 1 grade from baseline was 60.0% at Week 4, 67.3% at Week 12, 84.4% at Week 24, and 83.6% at Month 12. The percentage of patients with an improvement of ≥ 2 grades was 23.2% at Week 4, 29.9% at Week 12, 50.0% at Week 24, and 53.3% at Month 12.

### Change in severity of ocular signs and symptoms

**Fig. 3:**
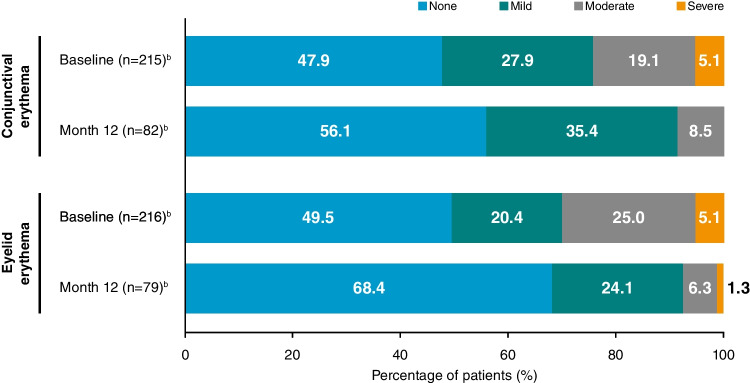
Severity of conjunctival erythema and eyelid erythema at baseline and Month 12 following the initiation of CsA 0.1% CE.^a^ ^a^Significant improvement from baseline to Month 12 (*P*< 0.001).^ b^Number of patients with conjunctival erythema or eyelid erythema data available/reported at study visit. CE, cationic emulsion; CsA, ciclosporin A

**Fig. 4:**
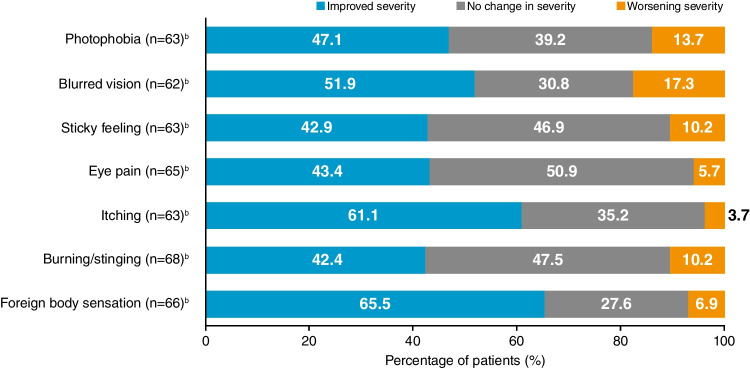
Change in severity of subjective symptoms at Month 12 following the initiation of CsA 0.1% CE.^a^ ^a^Significant improvements in all subjective symptoms from baseline to Month 12: photophobia (*P* = 0.015), blurred vision (*P* = 0.001), sticky feeling (*P* = 0.004), eye pain (*P* < 0.001), itching (*P* < 0.001), burning/stinging (*P* = 0.001), foreign body sensation (*P* < 0.001). ^b^Number of patients with subjective symptom data available/reported at Month 12. CE, cationic emulsion; CsA, ciclosporin A

At Month 12, the severity of eyelid erythema and conjunctival erythema was significantly reduced compared with baseline (*P* < 0.001; Fig. 3). Subjective symptoms were also significantly reduced in severity at Month 12, from baseline, following the initiation of CsA 0.1% CE, including photophobia (*P* = 0.015), blurred vision (*P* = 0.001), sticky feeling (*P* = 0.004), eye pain (*P* < 0.001), itching (*P* < 0.001), burning/stinging (*P* = 0.001), and foreign body sensation (*P* < 0.001) (Fig. 4). Mean TBUT was significantly increased from Week 4 (*P* = 0.0199) through to Month 12 (*P* < 0.001) compared with baseline. At Month 12, the mean TBUT was increased by 2.76 (SD 4.94) seconds. Changes in Schirmer’s test and VA were not statistically significant at Month 12.

### Physician and patient assessments

**Fig. 5:**
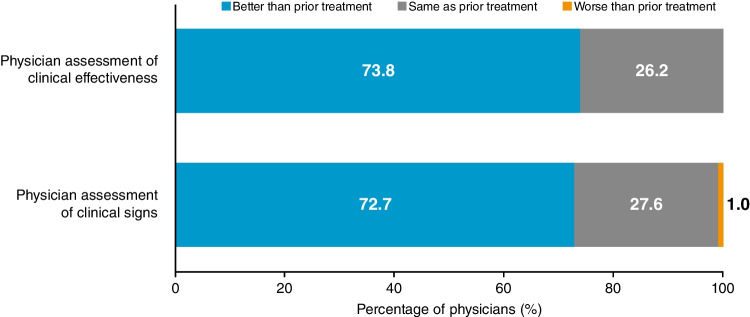
Physician assessment of clinical effectiveness and clinical signs with CsA 0.1% CE treatment after 12 months, compared with prior treatment. CE, cationic emulsion; CsA, ciclosporin A

Physicians reported clinical effectiveness with CsA 0.1% CE treatment to be either better than (73.8%) or the same as (26.2%) prior medication at Month 12. Most physicians (72.7%) also considered clinical signs to be improved with CsA 0.1% CE treatment at Month 12 (Fig. 5). Overall, 77.0% of patients and 80.4% of physicians reported tolerability with CsA 0.1% CE to be "good" or "very good" at Month 12.

Tolerability, as evaluated by the physician, was rated “good” or “very good” in 86.8% of patients at Month 12. The proportion of patients with “poor” tolerability, as evaluated by the physician, was only 2.8% at Month 12. In comparison, tolerability, as evaluated by the patient, was rated “good” or “very good” in 81.0% of patients at Month 12. The proportion of patients with “poor” tolerability, as evaluated by the patient, was 4.3% at Month 12.

### Discontinuation from study treatment

In total, 40 participants (16.9%) discontinued CsA 0.1% CE treatment during the study period. The reasons for discontinuation of CsA 0.1% CE treatment included insufficient control of keratitis or DED with prior medication (6 patients), poor local tolerance (11 patients), other reasons (10 patients), AEs (3 patients), and personal reasons (13 patients). Reasons for discontinuation were not mutually exclusive. No discontinuations were due to poor compliance or the progression of keratitis or DED during the study period.

Of the patients who discontinued CsA 0.1% CE, the majority did not receive corticosteroids at, or after, baseline. Only one patient who received corticosteroids prior to baseline (of which there were only 7 in total in Germany) failed to tolerate CsA 0.1% CE.

### Safety assessments

Overall, 112 AEs were reported during the study period. The majority were mild (52) or moderate (41), and 17 were severe (with data missing for two AEs). Of the 112 AEs, 37 were considered to be treatment related (Table 3), mainly eye irritation, and 14 were considered to be serious, including two that were deemed to be treatment related (back pain and thyroid disorder). The majority (75.7%) of treatment-related AEs resolved or were resolving by the end of the study period (28/37), one patient recovered but experienced sequelae, and 9 had not resolved. The majority (69.3%) of non-treatment related AEs also resolved or were resolving by the end of the study period (52/75), 11 had not resolved, and two AEs were fatal (intestinal infarction and malignant lung neoplasm), with 10 having the outcome unknown or missing data.

**Table 3:** Treatment-related AEs reported in Germany during the 12-month study period

Treatment-related AE	Number of events
**Ocular AEs**	
Cataract	2
Conjunctival hemorrhage	1
Dry eye	2
Eye discharge	1
Eye irritation	17
Eyelid margin crusting	1
Lacrimation increased	1
Ocular hyperemia	1
Pain	2
Vision blurred	2
**Other AEs**	
Dizziness	1
Headache	1
Back pain^a^	1
Nausea	1
Skin ulcer	1
Swelling face	1
Thyroid disorder^a^	1

^a^Assessed as a serious treatment-related AE required hospitalization

*AE*, adverse event

## Discussion

This sub-analysis of patients who were enrolled in the PERSPECTIVE study from Germany examined the effectiveness, tolerability, and safety of CsA 0.1% CE treatment in a real-world setting. The inclusion and exclusion criteria were reflective of the patient group defined in the approved CsA 0.1% CE label [5], and all study medications (CsA 0.1% CE, ATs, or corticosteroids) were prescribed in accordance with local healthcare arrangements. No major differences were observed in patient demographics between the overall study population (across Europe) and this sub-analysis (in Germany), with only the proportion of females being slightly lower in the German cohort (69.9% versus 75.9%). Only 7 patients (2.97%) were receiving concomitant corticosteroids at baseline (six using dexamethasone and one using fluorometholone). This is fewer than in the overall study population, where 7.4% of patients initiated corticosteroids in addition to CsA 0.1% CE at the baseline visit, and 8.5% were prior corticosteroid users who were expected to continue corticosteroids in addition to CsA 0.1% CE. No patients initiated corticosteroids in the German cohort, whereas 5.9% of patients in the overall study population initiated corticosteroids after the baseline visit.

The outcomes reported are similar to those reported for the overall study population [5]. These results are also consistent with reports from RCTs showing a significant and clinically meaningful reduction in corneal surface damage and improvement in ocular signs and symptoms of patients with severe keratitis and DED following treatment with CsA 0.1% CE [11–15]. The PERSPECTIVE study was designed to reflect real-world clinical practice, and, in contrast to conventional RCTs, no washout period was required prior to initiating treatment with CsA 0.1% CE [5]. Therefore, patients were permitted to continue concomitant treatment with corticosteroids and/or ATs when treatment with CsA 0.1% CE was initiated. As a consequence, these data reflect ophthalmology clinical practice in Germany, where patients may be prescribed multiple therapies and may also use over-the-counter medications. This approach is also consistent with the German Ophthalmology Society (DOG) and TFOS DEWS II guidelines [4, 8], as well as recent clinical consensus papers [18, 24], which have noted the anti-inflammatory and immunomodulatory effects of CsA treatment and recommended that such therapies should be used in conjunction with ATs and when AT therapy alone no longer provides adequate control [4, 8, 18, 24].

Despite potential differences in treatment approaches and/or assessment of DED (for inclusion and monitoring) across countries and healthcare settings, baseline clinical characteristics show only slight differences between this sub-analysis in Germany and the overall primary study population. To note, almost half of patients (42.9%) were using ATs at least once and up to three times a day in the overall study population, whereas this was 49.4% of patients in the German cohort. However, this may not be a confounding factor on the outcomes — while ATs may help in providing lubrication at the ocular surface, ATs do not address the underlying inflammatory pathways and subsequent ocular surface aberrations that drive the vicious cycle of DED and progressive disease [2, 4]. In addition, CFS score at baseline was typically graded at II or III, with a slightly larger proportion of patients (74.4%) in this German cohort than the overall study population (65.6%). This suggests that ophthalmologists, especially in Germany, select patients for topical CsA 0.1% CE therapy at an earlier disease stage compared with the inclusion criteria generally used in RCTs (CFS score IV or V) [11, 13, 14]. However, no formal thresholds have been defined regarding DED severity in relation to CFS score, and the patient’s perception of symptom severity might influence the grading attributed by the diagnosing physician or be conflicting with clinical signs.

Equally, it is important to note that with a real-world study design, some patients may receive topical corticosteroids during treatment with CsA 0.1% CE, and this could account for some of the anti-inflammatory effects and efficacy outcomes within the study. However, despite this potential bias, minimal corticosteroid use was reported throughout the 12-month time period in the German cohort. In the FAS, no patients initiated corticosteroids at the baseline visit in addition to CsA 0.1% CE, and only 7 patients in total were already receiving corticosteroids when they attended their baseline visit (and were expected to continue to do so). Throughout the study, four patients had corticosteroid use reported at Week 4, two at Week 12, one at Week 24, and one at Month 12. The number of patients receiving and initiating corticosteroids was fewer than that observed in the PERSPECTIVE study from the UK, but slightly more than that observed in Norway. Due to the small sample size, further sub-analyses were not carried out to account for the effect of concomitant use of corticosteroids.

The results of this sub-analysis support previous real-world study publications confirming the efficacy of CsA 0.1% CE in the treatment of severe keratitis and DED [5, 16, 21] and also provide country-level insights into real-life practice patterns and outcomes that ophthalmologists and patients may expect with this treatment in their healthcare setting in Germany.

In a real-world clinical practice setting, patients in Germany who were diagnosed with severe keratitis and DED benefited from CsA 0.1% CE treatment [5]. At Month 12, most patients (81.6%) experienced reductions in CFS score compared with baseline. Significant reductions in mean CFS score were observed from Week 4 and maintained throughout the 12-month study period (*P* < 0.0001), suggesting that the majority of patients treated with CsA 0.1% CE in clinical practice experience and maintain ocular surface improvements over a prolonged period. This change in CFS was observably different in the German cohort than the overall study population (both FAS) at Week 24 and Month 12, where the mean CFS score was 0.96 and 0.85, respectively, compared with 1.24 and 1.10. In a previous study, patients treated with CsA 0.1% CE for 12 months who experienced improvements in CFS scores sustained these improvements following treatment discontinuation [15]. Given the study design and typical bridging approaches with topical CsA in Germany, this is probably indicative of routine clinical practice in Germany and suggests that the majority of patients treated with CsA 0.1% CE may maintain ocular surface improvements over a prolonged period.

Consistent with the results reported in the primary analysis, patients treated with CsA 0.1% CE in Germany showed significant (*P* ≥ 0.01) improvements in the severity of clinical signs and ocular symptoms from Week 4, which were sustained through to Month 12 [5]. Physicians rated treatment effectiveness and clinical signs as better with CsA 0.1% CE compared with prior treatments, and both physicians and patients regarded tolerability with CsA 0.1% CE treatment to be "very good", "good", or "satisfactory". All of these results show commonality with the primary analysis. However, in contrast to the findings reported for the overall study population, changes in VA were not significant in the cohort of patients with DED who were treated with CsA 0.1% CE in Germany [5]. In addition, changes in the Schirmer’s test were neither significant in the overall population nor in the German cohort [5].

It is well established that a proportion of patients with DED seen by ophthalmologists in clinical practice will exhibit conflicting signs and symptoms, and this seems congruent with the population observed in the PERSPECTIVE study [5]. This may be of particular note in patients with CFS score of II (“mild”) or III (“moderate”). Additional sub-analyses to examine the change in ocular signs and symptoms by baseline CFS scores would be of value to provide further data regarding the key signs/symptoms that may have prompted decisions to initiate CsA 0.1% CE therapy and the treatment outcomes achieved in each of these subgroups.

Safety and tolerability data were consistent with the known safety profile of CsA 0.1% CE [5, 11–14]. There were no noticeable discoveries in the AEs reported in this German sub-analysis. Despite reports of some patients experiencing tolerability issues with CsA 0.1% CE, only 11 patients discontinued CsA 0.1% CE in the German cohort due to poor local tolerance (4.7% of the FAS – two by Week 4, three by Week 12, two by Week 24, and four by Month 12), which was higher than reported for the overall study population (6.6% of the FAS) but similar to the rate of discontinuation due to ocular treatment-related AEs reported in other studies of CsA 0.1% CE in the treatment of severe keratitis and DED (9.3–10.4%) [11, 14, 25]. It could be hypothesized that the lower proportion of patients with tolerability issues in the German cohort could be due to the difference in AT and/or corticosteroid use prior to and with CsA 0.1% CE treatment. Overall, the safety profile and treatment benefit reported in real-world clinical studies suggest a favorable benefit–risk ratio for CsA 0.1% CE in the treatment of severe keratitis and DED [5, 16, 21].

This sub-analysis includes one cohort from the overall study population that was enrolled in the PERSPECTIVE study, and therefore, the number of patients is smaller than those included in the primary analysis, which may impact the results. Although the study was prospective, it was also observational, which is associated with limitations, such as a lack of a control or placebo arm for comparison. The study population is heterogenous as it was based on clinical practice, and clinical judgement was used to determine which patients were suitable for treatment with CsA 0.1% CE, according to the approved European label [20]. Although the population is heterogeneous, as a country-level analysis, the population is assumed to be more homogenous than the overall study population. It also reflects the range of signs, symptoms, and effects that patients with severe keratitis and DED might experience on a day-to-day basis rather than a defined population with specific characteristics as seen in RCTs, and therefore, it is consistent with routine clinical practice in Germany.

## Conclusion

A country-level sub-analysis of the PERSPECTIVE study data from ophthalmology clinics in Germany shows similar outcomes to those reported for the overall study population and is indicative of the treatment results that ophthalmologists may expect to see with CsA 0.1% CE treatment in real-life clinical practice. CsA 0.1% CE provided significant reductions in the severity of keratitis and DED signs and symptoms, which were demonstrated from Week 4 and maintained through to Month 12. Treatment with CsA 0.1% CE was generally well tolerated.

## Data Availability

The datasets generated and/or analyzed during the study are available from the corresponding author on reasonable request.

## Declarations

### The PERSPECTIVE German study group

Members of the PERSPECTIVE German study group were the following: Christophe Deuter (Augenklinik des Universitätsklinikums Tübingen, Tübingen), Katrin Engelmann (Klinikum Chemnitz gGmbH, Chemnitz), Nicole Eter (Universitaets-Augenklinik Muenster, Muenster), Gerd Geerling (Augenklinik des Universitätsklinikums Düsseldorf, Düsseldorf), Andrea Hoeck (Universitäts-Augenklinik Bonn, Bonn), Kai Januschowski (Mount Saint Peter Eye Clinic, Trier; University of Tübingen, Tübingen), Daniel Kampik (Department of Ophthalmology, University Hospitals Würzburg, Würzburg), Karsten Klabe (Internationale Innovative Ophthalmochirurgie, Düsseldorf), Thomas Kohnen (Klinikum der Johann Wolfgang Goethe-Universitaet Frankfurt, Frankfurt), Lothar Krause (Städtisches Klinikum Dessau, Dessau-Rosslau), Friedrich E Kruse (Augenklinik des Universitetsklinikums Erlangen, Erlangen), Ines Lanzl (Chiemsee Augen Tagesklinik, Prien), Katrin Lorenz (Universitaetsmedizin der Johannes Gutenberg-Universitaet Mainz, Mainz), Elisabeth M. Messmer (Department of Ophthalmology, Ludwig Maximilians University, Munich), Arthur Mueller (Klinik für Augenheilkunde Universitätsklinikum Augsburg, Augsburg), Thomas Neuhann (MVZ Professor Neuhann, GmbH, Munich), Berthold Seitz (Universitaetsklinikum des Saarlandes, Homburg), Martin Spitzer (Universitätsklinikums Hamburg-Eppendorf, Hamburg), Hagen Thieme (Otto-von-Guericke-Universität, Medizinische Fakultät, Universitätsklinikum Magdeburg A.ö.R., Magdeburg), Christian van Oterendorp (University Medical Center Göttingen, Department of Ophthalmology, Göttingen), Manfred Zierhut (Augenklinik des Universitätsklinikums Tübingen, Tübingen). For the full list of investigators, please refer to the primary study article [5].
